# Proprotein Convertase Subtilisin/Kexin Type 9 Inhibitors in Patients Following Acute Coronary Syndromes: From Lipid Lowering and Plaque Stabilization to Improved Outcomes

**DOI:** 10.3390/jcm13175040

**Published:** 2024-08-25

**Authors:** Kyriakos Dimitriadis, Nikolaos Pyrpyris, Panagiotis Iliakis, Eirini Beneki, Eleni Adamopoulou, Aggelos Papanikolaou, Dimitrios Konstantinidis, Christos Fragkoulis, Anastasios Kollias, Konstantinos Aznaouridis, Konstantinos Tsioufis

**Affiliations:** 1 First Department of Cardiology, School of Medicine, National and Kapodistrian University of Athens, Hippokration General Hospital, 11527 Athens, Greece; npyrpyris@gmail.com (N.P.); panayiotisiliakis@gmail.com (P.I.); e.beneki@hotmail.com (E.B.); 99elenaadam@gmail.com (E.A.); agepap25@otenet.gr (A.P.); kon_dimitris@hotmail.com (D.K.); christosfragoulis@yahoo.com (C.F.); conazna@yahoo.com (K.A.); ktsioufis@gmail.com (K.T.); 2 Hypertension Center STRIDE-7, Third Department of Medicine, School of Medicine, National and Kapodistrian University of Athens, Sotiria Hospital, 15772 Athens, Greece; taskollias@gmail.com

**Keywords:** acute coronary syndrome, lipids, PCSK9 inhibitors, atherosclerosis, dyslipidemia, myocardial infarction, statins

## Abstract

Lipid lowering, with the use of statins after an acute coronary syndrome (ACS), is a cornerstone, well-established strategy for the secondary prevention of ischemic events in this high-risk cohort. In addition to the positive effect on lipid levels, statins have also been linked to improved atherosclerotic plaque characteristics, such as plaque regression and inflammation reduction, associated with the extent of reduction in LDL-C. The recent emergence of PCSK9 inhibitors for the management of dyslipidemia and the more extensive lipid lowering provided by these agents may provide better prevention for ACS patients when initiated after the ACS event. Several trials have evaluated the immediate post-ACS initiation of PCSK9 inhibitors, which has shown, to date, beneficial results. Furthermore, PCSK9 inhibitors have been linked with positive plaque remodeling and associated mortality benefits, which makes their use in the initial management strategy of such patients appealing. Therefore, in this review, we will analyze the rationale behind immediate lipid lowering after an ACS, report the evidence of PCSK9 inhibition immediately after the ACS event and the available data on plaque stabilization, and discuss treatment algorithms and clinical perspectives for the use of these agents in this clinical setting.

## 1. Introduction

The global prevalence of dyslipidemia has been increasingly rising [1]. Although it is established knowledge that dyslipidemia is associated with an elevated risk of cardiovascular disease (CVD), it is of great importance to highlight this heightened association with plasma low-density lipoprotein cholesterol (LDL-C) levels, which was the eighth leading death factor in 2019 [2]. The two most common types of dyslipidemias are primary (or hereditary or familial) [3] and secondary in the setting of other cardiometabolic conditions, such as diabetes mellitus type II (DM2), obesity, unhealthy lifestyle, and hypertension [4]. Regarding the latter, which is more common and frequent, when combined with increased levels of triglycerides and a decreased level of high-density lipoprotein cholesterol (HDL-C), it has been defined as atherogenic dyslipidemia, which is highly associated with elevated CVD risk and CVD mortality [5]. Although the need for LDL-C level lowering is strongly emphasized in both primary and secondary prevention [6,7], statin intolerance, inertia-to-treatment, and socioeconomic burden lead to very low LDL-C control [8,9], especially in high-cardiovascular (CV)-risk patients [10]. Particularly interesting is the fact that even in patients with recurrent events, LDL-C targets may be suboptimal. This has been shown by Ciliberti et al. [11] in patients with recurrent acute myocardial infarction after myocardial infarction with non-obstructed coronary arteries (MINOCA) diagnosis, reporting that only 4.5% had LDL-C levels below 55 mg/dL. This further underlines the potential underestimation of cardiovascular risk by physicians, along with the aforementioned limitation, in certain patient populations.

This unmet need moved research further in order to explore novel lipid-lowering agents that are involved in the hepatic metabolism of LDL-C and explore possible pharmaceutical targets of the multi-involved cascades [5,6]. Proprotein convertase subtilisin/kexin type 9 (PCSK9) is a serine protease that is mainly expressed in hepatic cells, whose main mechanism is the binding of LDL-C receptor (LDL-R) on the cellular membrane, leading to the degradation of LDL-R by endocellular lysosomes, reducing LDL-C intake, and, therefore, leading to a consequent increase in LDL-C plasma levels [12]. This mechanism is targeted and blocks PCSK9 inhibitors, resulting in LDL-R upregulation and a decrease in LDL-C plasma levels. A large amount of evidence regarding the safety, efficacy, and tolerability of these agents became quickly available. In one of the first randomized control trials (RCT), Sullivan et al. [13] showed both high tolerability and efficacy in statin-intolerant patients. Similarly, DESCARTES, one of the first phase III trials with evolocumab, also reported significant LDL-C reductions, irrespective of the anti-lipid regimen the patients were receiving [14]. Given the recently accumulated data in acute coronary syndromes (ACS), this review aims to analyze post-ACS lipid-lowering rationale and delve into the early utilization of PCSK9 inhibitors in high-risk patients, highlighting the simultaneous importance of “the lower-the better” and “the sooner-the better” guide toward decreasing cardiovascular mortality [15].

## 2. Rationale for Post-ACS Lipid Lowering

It is very important to initiate lipid-lowering treatment after ACS, as it is established knowledge from RCTs, as well as cohort studies, that remarkable LDL-C reduction is strongly associated with improvements in mortality and nonfatal major adverse cardiovascular events (MACEs) [16]. The 2019 European Society of Cardiology/European Atherosclerosis Society (ESC/EAS) Guidelines for the management of dyslipidemias suggested a decrease in LDL-C of 1.4 mmol/L or lower than 55 mg/dL, as well as a more than 50% reduction in LDL-C since baseline (Level of Recommendation I, Level of Evidence A) [6]. In the last few decades, a vast series of RCTs, cohorts, and meta-analyses have been conducted, demonstrating the importance of early and intensive LDL-C lowering after an ACS. The most studied drugs are statins, which mainly act by inhibiting 3-hydroxy-3-methylglutaryl coenzyme A (HMG-CoA) reductase enzyme, leading to a decrease in hepatic cholesterol production [17].

The myocardial ischemia reduction with aggressive cholesterol lowering (MIRACL) study was one of the first multicenter RCTs to assess the impact of statin therapy in recurrent ischemia; 3086 patients hospitalized with ACS were enrolled and randomized to either 80 mg of atorvastatin or a matching placebo and followed up for 16 weeks [18]. The investigators documented a decreased symptomatic ischemia risk compared to the placebo group (6.2% vs. 8.4%; RR, 0.74; 95% CI, 0.57–0.95; *p* = 0.02). In 2004, Cannon et al. conducted one of the first RCTs evaluating the effect of intensive versus standard statin therapy on mortality after an ACS. A total of 4162 patients were enrolled and randomized to standard lipid-lowering treatment (pravastatin 40 mg) or intensive treatment (atorvastatin 80 mg), demonstrating a statistically significant association between intensive statin treatment and a reduction in MACEs (composite endpoint of both fatal and nonfatal MACEs) linearly connected to LDL-C level reductions [19]. Similarly, LaRosa et al. evaluated the efficacy of an intensive lowering strategy in patients with chronic CVD [20]. A total of 10,001 symptomatic patients with chronic CVD and LDL-C levels < 130 mg/dL were randomized to daily statin treatment with 80 mg of atorvastatin (intensive) and 10 mg of atorvastatin (standard). Although there was no between-group difference regarding overall mortality, the primary endpoint (of both fatal and non-fatal MACEs) occurred in 8.7% of the intensive arm compared to 10.9% of the standard arm, and the intensive lipid-lowering strategy was associated with a 22% relative risk reduction (HR, 0.78; 95% CI 0.69 to 0.89; *p* < 0.001).

In a meta-analysis of 26 RCTs and 170,000 patients regarding the comparison of lipid-lowering treatment with statins vs. placebo and the comparison of intensive vs. standard lipid-lowering treatment with statins, the aforementioned findings were further supported [21]. This landmark study demonstrated that all-cause mortality was reduced by 10% per 1.0 mmol/L of LDL-C level decrease (RR 0.90, 95% CI 0.87–0.93; *p <* 0.0001). Regarding intensive vs. standard statin treatment, intensive treatment was associated with a significant reduction in major cardiovascular events by 15% (95% CI 11–18; *p* < 0.0001), death due to myocardial infarction (MI) or nonfatal MI by 13% (95% CI 7–19; *p* < 0.0001), and coronary revascularization by 19% (95% CI 15–24; *p* < 0.0001) [21]. With respect to statin initiation timing, a meta-analysis by Navarese et al. evaluated the administration of statins before or after percutaneous coronary intervention (PCI) in patients with ACS vs. no or a low dose of statin regarding all-cause mortality and incidence of new MI at 30 days after the initiation [22]. They included 20 RCTs and 8750 patients, demonstrating that earlier statin initiation was significantly associated with a lower risk of new MI and overall mortality at 30 days, highlighting the efficacy of the “the-sooner-the-better” strategy in patients hospitalized for ACS [22].

IMPROVE-IT was the first double-blind RCT that assessed the safety and efficacy of ezetimibe as an on-top statin treatment in patients hospitalized for ACS [23]. Cannon et al. enrolled 18,144 patients with ACS and LDL-C levels of 50–100 mg/dL who were randomized either to the intensive treatment arm (40 mg of simvastatin + 10 mg of ezetimibe or to the standard treatment arm (40 mg of simvastatin). They demonstrated that the dual-combination regimen led to greater LDL-C reduction (53.7 mg/dL vs. 69.5 mg/dL, *p* < 0.001), as well as a significant reduction in MACEs (rate for the primary endpoint at 7 years was 32.7% vs. 34.7%, HR, 0.936; *p* = 0.016). IMPROVE-IT [23] was, therefore, mentioned in the 2019 ESC guidelines, leading to the recommendation of intensive lipid-lowering treatment, with a combination of high-intensity statin and ezetimibe, in post-ACS patients [6].

Statin intolerance is a significant factor contributing to suboptimal LDL-C level control, especially in patients with established CVD [24]. In GAUSS-3, PCSK9 inhibitors were evaluated, in a randomized setting and in comparison to ezetimibe, in patients with documented statin intolerance. Nissen et al. demonstrated that evolocumab led not only to greater LDL-C reduction after 24 weeks compared to ezetimibe (mean absolute change with ezetimibe −31.2 mg/dL, mean absolute change with evolocumab −102.9 mg/dL (*p* < 0.001), but also to fewer muscle symptoms [25]. The aforementioned evidence leads to the optimal assessment of cardiovascular risk in lipid-lowering therapy and toward up-titrating and adding a second or even a third lipid-lowering drug [26]. Up-titration of lipid-lowering treatment is essential, and its effect is even greater in patients at high or very high cardiovascular risk, as a metanalysis of 14 trials, including 83,660 adults, reported that the addition of ezetimibe or PCSK9 inhibitors was associated with a reduction in stroke and nonfatal MI [27].

Lipid-lowering treatment also has an important role in plaque stabilization through plaque volume reduction and fibrous cap thickening [5,28,29]. Regarding plaque stabilization, the ESTABLISH trial demonstrated a significant plaque volume (PV) decrease with atorvastatin compared to the control (−13.1 +/− 12.8% vs. +8.7 +/− 14.9%, *p* < 0.0001) [30]. In JAPAN-ACS, a prospective, multicenter RCT, Hiro et al. evaluated the effect of 4 mg of pitavastatin compared to 20 mg of atorvastatin regarding intracoronary imaging-evaluated change in non-culprit PV in patients with ACS. They reported an equivalent significant reduction in PV in both arms (−16.9 +/− 13.9% vs. −18.1 +/− 14.2%, *p* = 0.5) [31]. Similarly, when rosuvastatin and atorvastatin were compared regarding LDL-C reduction and modification of PV in ACS patients, an equivalence between the two arms was documented [32]. Sharing the randomized design of IMPROVE-IT, PRECISE-IVUS compared the effect of atorvastatin plus ezetimibe vs. atorvastatin alone, on intravascular ultrasound-assessed PV reduction in 202 ACS patients, at baseline and 12 months after the ACS. The study showed that the dual treatment led to greater reductions in LDL-C levels (63.2 ± 16.3 mg/dL vs. 73.3 ± 20.3 mg/dL; *p* < 0.001), as well as a significant decrease in PV (−1.4% vs. −0.3%, *p* = 0.001) [33].

Regarding fibrous cap thickness, EASY-FIT randomized 17 patients with unstable angina to either 20 mg or 5 mg of atorvastatin and evaluated them via optical coherence tomography (OCT) at baseline and after 12 months. Intensive atorvastatin treatment was significantly associated with a greater increase in fibrous cap thickness, which was correlated with an absolute reduction in LDL-C levels (R = −0.450; *p* < 0.001) [34]. Similar results were reported in the ESCORT trial, a prospective, randomized, active-controlled trial, in which 53 patients with ACS were assigned to 4 mg of pitavastatin from baseline (early arm) or 4 mg of pitavastatin 3 weeks after the procedure (late arm). Patients were assessed with OCT at baseline, 3 weeks, and 36 weeks after the procedure. The early treatment arm documented a significantly greater increase in fibrous cap thickness during the first 3 weeks of follow-up and a further increase at 36 weeks of follow-up [35]. Finally, the addition of ezetimibe to fluvastatin, compared to fluvastatin alone, in 57 patients with ACS, led to a significant increase in OCT-evaluated fibrous cap thickness at 9 months from baseline in the combination treatment arm compared to single fluvastatin treatment (0.08 ± 0.08 mm vs. 0.04 ± 0.06 mm, *p* < 0.001) [36].

## 3. Studies of PCSK9 after ACS

FOURIER and ODYSSEY were the landmark RCTs in large-scale population samples with known CVD [37,38] that supported the safety and efficacy of PCSK9 inhibitors in improving cardiovascular death and nonfatal MACEs, thus establishing PCSK9-related suggestions in the recent 2019 ESC/EAS Guidelines [6].

Regarding secondary prevention, FOURIER was the first double-blind RCT that evaluated the safety and efficacy of evolocumab in 27,564 patients with established CVD and LDL-C levels ≥ 70 mg/dL under statin therapy. Sabatine et al. demonstrated that evolocumab led to a significant reduction in LDL-C levels (from a mean of 92 mg/dL at baseline to a mean of 30 mg/dL at 48 weeks), accompanied by a significant reduction in the primary composite endpoint of cardiovascular death, MI, stroke, hospitalization for unstable angina, or need for coronary revascularization by 15% (HR 0.85, 95% CI 0.79–0.92] at 2.2 years of follow-up [37]. In a prespecified secondary analysis of the FOURIER trial, the investigators included 22,320 patients with a recent MI prior to randomization. It is important to note that patients with MI within 4 weeks prior to randomization were, per protocol, excluded from the FOURIER trial. They demonstrated that, in patients with recent MI, evolocumab led to a significant decrease in the risk of the composite primary endpoint (cardiovascular death, MI, stroke, hospitalization for unstable angina, or coronary revascularization) by 19% (HR 0.81; 95% CI, 0.70–0.93) [39]. Moreover, the beneficial effect of evolocumab was consistent, regardless of the type of MI, with the greater reduction being observed in patients whom troponin levels were ≥10 × the upper limit of normal values (34%, HR, 0.66; 95% CI, 0.56–0.77; *p* < 0.001) [40]. Further aiming to evaluate the “the lower—the better” strategy, Giugliano et al., in a subsequent secondary analysis, evaluated the relationship between the achieved LDL-C levels at follow-up and the incidence of the primary end-point. The investigators reported a monotonic relationship between achieved LDL-C levels and composite primary endpoint (death and non-fatal MACEs), even in LDL-C levels lower than 7.7 mg/dL (0.2 mmol/L), suggesting its safety and even indicating a further reduction in LDL-C levels compared to the current guidelines’ suggestions [41]. Moreover, when patients of the FOURIER trial were stratified according to renal function, evolocumab’s effect on both LDL-C-lowering and -improving MACEs was similar across the renal function spectrum; however, the numerical reduction was greater in patients with more advanced kidney disease [42]. Finally, with respect to cardiovascular mortality, evolocumab was found to have a secondary prevention effect not only on patients with coronary artery disease but in all vascular territories, as it was shown that the addition of evolocumab, on top of maximally tolerated statin therapy, significantly reduced peripheral arterial events by 16% (HR 0.84, 0.75–0.95) at 12 months since initiation [43].

ODYSSEY OUTCOMES was the first double-blind RCT, designed to resemble the FOURIER setting, that evaluated the safety and efficacy of alirocumab in 18,924 patients previously hospitalized for acute MI or unstable angina and had LDL-C levels ≥ 70 mg/dL under statin therapy. All patients were randomized to either alirocumab or a placebo [38]. Similarly to alirocumab and showing the first evidence of a drug-class effect, evolocumab was associated with a significant reduction in LDL-C levels (from a mean of 92 mg/dL at baseline to a mean of 48 mg/dL at 12 months), accompanied by a significant reduction in the primary composite endpoint (cardiovascular death, MI, stroke, or hospitalization for unstable angina) by 15% (HR 0.85, 95% CI 0.78–0.93] at a 2.8-years follow-up period [38]. In a prespecified analysis of ODYSSEY OUTCOMES, including patients with a previous MI (3633 patients, 19.2% of the total ODYSSEY OUTCOMES population), it was shown that the 4-year risk of fatal and nonfatal MACEs was higher among those with a previous MI, while the addition of alirocumab led to a greater absolute risk reduction when compared with patients without a history of previous MI (nonfatal MACEs: 1.91% vs. 1.42%; death: 1.35% vs. 0.41%) [44]. Alirocumab was also associated with a very high rate of LDL-C control, leading to 94.6% of the patients having LDL-C levels lower than 1.4 mmol/L (or 55 mg/dL), thus achieving the LDL-C levels goal according to the 2019 European guideline [45]. Moreover, similarly to evolocumab, alirocumab was associated with reductions in polyvascular disease (coronary, peripheral, and cerebrovascular)-related MACEs and mortality, with the absolute risk reduction being 1.4% (95% CI: 0.6% to 2.3%), 1.9% (95% CI: −2.4% to 6.2%), and 13.0% (95% CI: −2.0% to 28.0%), respectively [46]. Regarding apolipoprotein A and apolipoprotein B metabolism and their association with cardiovascular outcomes after PCSK9 initiation in ACS, it is demonstrated that reduction in the total fatal and nonfatal MACEs was greater at higher levels of the aforementioned proteins, and moreover, the achievement of apolipoprotein B levels lower than 35 mg/dL decreased the lipoprotein-derived residual risk after ACS [47]. Finally, it is noteworthy that, although there are some racial differences regarding dyslipidemia prevalence and cardiovascular risk globally, it was found that both evolocumab and alirocumab were safe, tolerant, and equally effective at reducing LDL-C levels and MACEs in Asian populations [48,49]. These results were also verified in another RCT, performed in Japan, that evaluated the effect of evolocumab, on top of statin therapy, in patients with MI and documented its beneficial effect on reducing both LDL-C and lipoprotein (a) levels 4 weeks after treatment initiation [50].

## 4. PCSK9 Inhibitors Immediately after an Acute Coronary Syndrome

As evidence for the safety and efficacy of PCSK9 inhibition became available, particular interest was drawn into the early administration of PCSK9 inhibitors, especially after an ACS. Immediate LDL-C reduction was proven to provide survival benefits and a reduction in adverse outcomes in the early era of statin treatment, which finally resulted in the initiation of guideline-recommended intensive lipid lowering after MI with these agents. Thus, the quicker and greater lipid level reduction provided by PCSK9 inhibition could greatly alter outcomes and potentially become a cornerstone of treatment in patients following an acute event. In this context, several investigators aimed to analyze the safety and efficacy of PCSK9 inhibition early on after the MI in order to better understand how the addition of this agent affects lipid levels and adverse outcomes (Table 1).

EVOPACS [51] was one of the first studies that evaluated the early initiation of PCSK9 inhibitors after an ACS. Specifically, the investigators studied the in-hospital initiation of the agent. They enrolled 308 ACS patients who had not achieved the desirable LDL-C levels with statin treatment and were randomly assigned to either evolocumab or placebo, starting in-hospital and after 4 weeks, for a follow-up of 8 weeks. Most patients were statin-naïve (78.2%). LDL-C levels significantly decreased in the PCSK9-inhibitor arm, with a mean difference percentage of −40.7% (95% CI −45.2 to −36.2%; *p* < 0.001), while LDL-C levels below 70 mg/dL were achieved in 95.7% of the evolocumab group and 37.6% of the placebo group. No difference was noted in adverse events, both overall and cardiovascular.

VCU-AlirocRT [52] was another early PCSK9-inhibitor initiation trial, initiating alirocumab treatment at the time of the myocardial infarction (defined as within the first 24 h of presentation) in 20 patients with LDL > 70 mg/dL. Alirocumab significantly reduced LDL-C from baseline to 14 days by 64 mg/dL (−96, −47) compared with the placebo [+1 mg/dL (−25, +16)]. These effects were accompanied by a significant reduction in the PCSK9 levels. Despite there being numerically more adverse events in the PCSK9-inhibitor cohort, none was believed to be attributed to the medication. Of note, PCSK9 inhibitors reduced the levels of LDL-C rapidly, as at 72 h, there was a significant difference between the groups, with a mean LDL-C reduction of 20 mg/dL in the PCSK9 inhibitor arm and 4 mg/dL in the control arm.

Followingly, the EVACS I trial [53] included 57 patients with suboptimal LDL-C levels and randomized them to either receive one dose of evolocumab at the time of the acute coronary event or a placebo on top of standard therapy. A total of 60% of patients were previously on statins. LDL-C was found to decrease from baseline by day 1 in the evolocumab arm (91.5 ± 35 to 70.4 ± 27 mg/dL; *p* < 0.01) and was lower compared to placebo by the third day (*p* = 0.02). This difference remained significant throughout the in-hospital period and at the 30-day follow-up (*p* < 0.01). Notably, after adjustments, LDL-C was lower by an average of 28.6 mg/dL in the PCSK9 group at the time of the follow-up, while 65.4 and 80.8% achieved LDL-C targets, compared to 23.8 and 38.1% (based on European and American guidelines, respectively) in the placebo arm.

The same group also reported on the impact of PCSK9 inhibition in lipoprotein a [Lp(a)] levels in the early post-ACS period [54]. This EVACS I and EVACS II (still ongoing) sub-study included 75 ACS (both non-ST elevation MI and ST elevation MI and NSTEMI and STEMI) patients, who were treated in a similar to EVACS I manner with either evolocumab or placebo. In the placebo arm, there was a significant increase in Lp(a) from a baseline of 63 nmol/L) to discharge (80 nmol/L) and 30 days (82 nmol/L), which was, however, mostly driven by those with high Lp(a) (>75 nmol/L) upon admission (a change of 28% vs. 10%). On the contrary, those treated with PCSK9 had no significant difference in Lp(a) in the two aforementioned timepoints, thus concluding that the administration of PCSK9 inhibitor can limit the increase in Lp(a) in the post-MI period.

More data on Lp(a) were provided by Nakamura et al. [55] in association with plasma kinetics of PCSK9. Plasma PCSK9 can be mature or furin-cleaved, and Lp(a) mostly binds to the mature subtype. As shown in this investigation, PCSK9-inhibitor administration leads to early (by day 3) reduction in both PCSK9 types, while the incremental area under the curve for Lp(a) was significantly reduced in the evolocumab versus the control group. Notably, Lp(a) and both PCSK9 types were significantly increased from baseline to day 3 and returned to normal after 30 days, showcasing the potential benefit of early PCSK9 administration.

**Table 1:** Key studies comparing PCSK9 inhibitors to placebo in patients with acute coronary syndromes.

Study	Study Type, Year	PCSK9 Inhibitor	Participants (n)	Setting	Follow-Up	LDL-C Reduction from Baseline		LDL-C Difference at Follow-Up between Groups	Adverse Outcomes	Other Outcomes
						PCSK9i (mg/dL)	Control (mg/dL)			
EVOPACS [51]	RCT, 2019	Evolocumab—420 mg (in-hospital and week 4)	308 ACS patients (1:1)	ACS, suboptimal LDL-C	8 weeks	139.6 to 30.55	132.25 to 79.66	−40.7% (95% CI: −45.2 to −36.2; *p* < 0.001)	No difference between groups	LDL-C < 70 mg/dL; PCSK9i: 95.7% control: 37.6%
VCU-AlirocRT [52]	RCT, 2019	Alirocumab—150 mg (in-hospital)	20 ACS patients (1:1)	NSTEMI, LDL-C > 70 mg/dL on statin	14 days	91 to 28	98 to 90	NR	None related to treatment	No difference in hs-CRP
EVACS I [53]	RCT, 2020	Evolocumab—420 mg (in-hospital)	57 ACS patients	NSTEMI	30 days	91.5 to 35.9	89.6 to 64.5	Evolocumab arm LDL-C a mean of 28.6 mg/dL lower than placebo (*p* < 0.001)	None related to treatment	LDL-C at targets: PCSK9i: 65.4–80.8% control: 23.8–38.1% (*p* = 0.01)
Vavuranakis et al. [54]	RCT, 2022	Evolocumab—420 mg (in-hospital)	74 ACS patients	NSTEMI	30 days	NR	NR	NR	None related to treatment	Lp(a): PCSK9i: 49 to 44 nmol/L (*p* = NS) control: 64 to 82 nmol/L (*p* < 0.01)
EPIC-STEMI [56]	RCT, 2022	Alirocumab—150 mg (pre-PCI, 2 weeks, 4 weeks)	68 ACS patients	STEMI	6 weeks	114.85 to 29	110.98 to 50.27	−22.3% (95% CI: −31.1 to −13.5; *p* < 0.001).	None related to treatment	LDL-C at targets: PCSK9i: 92.1% control: 56.7%
Xu et al. [57]	Prospective, 2021	Evolocumab—140 mg (in-hospital and every 2 weeks)	334 ACS patients (96 PCSK9i vs. 238 control)	NSTEMI, suboptimal LDL-C	12 weeks	143 to 27	127.6 to 77.4	−41.8% (95% CI −45.0 to −38.5%; *p* < 0.001)	No significant differences	LDL-C <55 mg/dL PCSK9i: 90.6% control: 7.1%
Zhang et al. [58]	Retrospective, 2022	Evolocumab—140 mg (in-hospital and every 2 weeks	1654 ACS patients (414 PCSK9; 1150 Control)	ACS, suboptimal LDL-C	18 months	129.2 to 28.6	126 to 78.1	−42.48 (−40.51 to −44.45; *p* < 0.001)	Composite of ischemic events and mortality: PCSK9i: 8.2% vs. control: 12.4%; HR: 0.65; 95% CI, 0.45–0.95) No safety events	LDL-C < 55 mg/dL PCSK9i: 91.6% control: 10.7%
Hao et al. [59]	RCT, 2022	Evolocumab—140 mg (in-hospital and every 2 weeks	136 ACS patients	High-risk ACS, suboptimal LDL-C	3 months	136.9 to 22.4	136.2 to 49.1	−83.9% vs. −63.9%	MACE: PCSK9i: 88% vs. control: 24.6%; *p* = 0.015	LDL-C at targets: PCSK9i: 82.4% control: 22%

Abbreviations: RCT: randomized control trial; ACS: acute coronary syndrome; PCSK9: Proprotein Convertase Subtilisin–Kexin Type 9; PCSK9i: Proprotein Convertase Subtilisin–Kexin Type 9 Inhibitor; LDL-C: low-density lipoprotein—cholesterol; NSTEMI: non-ST elevation myocardial infarction; LP(a): Lipoprotein a; MACE: major adverse cardiovascular events; PCI: percutaneous coronary intervention; hs-CRP: highly sensitive C-reactive protein; HR: Hazard Ratio; CI: Confidence Interval.

The EPIC-STEMI study [56] provided more evidence of PCSK9’s early initiation after ACS, including only STEMI patients. All enrolled individuals were randomized to either alirocumab or control, with the first injection before PCI regardless of LDL-C levels and two follow-up doses at 2 and 4 weeks. The participants were followed up for a total of 6 weeks post-MI. The mean LDL-C was significantly decreased between the two cohorts by 72.9% in the alirocumab and 48.1% in the sham group (a mean difference of −22.3%; *p* < 0.001). Similarly to NSTEMI studies, 92.1% of alirocumab-treated patients achieved European guidelines targets, in comparison to 56.7% of controls, while PCSK9 inhibitor use was associated with a more rapid LDL-C decline in the first 24 h compared to the sham group (−0.01 mmol/L/hour; *p* = 0.03).

Regarding evidence in Asian cohorts, Xu et al. [57], in a prospective study, showed that the in-hospital administration of evolocumab in NSTEMI patients with suboptimal LDL-C levels, under statin treatment and compared to controls, leads to significant reductions in LDL-C at 8 and 12 weeks follow-up, with an approximately −79% change from baseline. Moreover, the mean difference at 12 weeks between controls and evolocumab-treated patients was significant (−41.8%; 95% CI: −45.0 to −38.5%; *p* < 0.001), while the percent of patients achieving LDL-C < 55 m/dL was significantly higher with PCSK9 inhibitors (90.6 vs. 7.1%; *p* < 0.001). Similarly, Zhang et al. [58] and Hao et al. [59], evaluating evolocumab after ACS in a similar population, also found significant reductions in lipid levels and no safety concerns, while both studies reported a reduced incidence of MACE after PCI in the evolocumab arm, showing an improved cardiovascular prognosis. As data became available from studies focusing on LDL-C reduction, several investigators, as will be described below, evaluated the effect of PCSK9 inhibitors on plaque characteristics. All trials found significant reductions in LDL-C levels, which were associated with plaque regression. This highlights the association of extensive lipid lowering with a benefit in plaque stabilization, which will be further analyzed in the following section.

Finally, a recent meta-analysis [60] including the available randomized trials comparing PCSK9 inhibitors versus placebo immediately after ACS showed that the LDL-C levels were significantly different, with a mean difference of −44.0 mg/100 mL (95% CI: −54.3 to −33.8; *p* < 0.001), as were Lp(a) (mean difference—24.0 nmol/L, 95% CI: −43.0 to −4.9; *p* = 0.01), total cholesterol (mean difference −49.2 mg/100 mL, 95% CI −59.0 to −39.3), apolipoprotein B (mean difference −33.3 mg/100 mL, 95% CI −44.4 to −22.1), and triglyceride (mean difference −19.0 mg/100 mL; 95% CI −29.9 to −8.2) levels. Thus, this data synthesis documented the effectiveness of these agents in significantly further reducing lipid levels compared to standard treatment.

Moving on from evidence from randomized trials, recently, Gargiulo et al. performed an analysis of real-world data from the AT-TARGET-TI registry on the early initiation of PCSK9 inhibitors after ACS. They showed that among 771 included patients, the addition of the PCSK9 inhibitor resulted in a significant drop of LDL-C from a median baseline level of 137 mg/dL to 43 mg/dL at the first lipid control, with 68.3% of patients having achieved LDL-C targets at this time point. Furthermore, through 11 months of follow-up, the investigators noted a stepwise lower risk of MACE, all-cause mortality, and ischemia-driven revascularization in the lower quartiles of LDL-C levels at the first lipid control and in patients with LDL-C levels lower than 55 mg/dL [61]. These data, along with evidence of high adherence (99.7%) in the same registry [62], highlight the benefit of early treatment after ACS in both clinical trials as well as real-world settings.

As analyzed thus far, in-hospital or early initiation of PCSK9 inhibitors in patients with ACS is a safe and efficient option, mostly tested in patients with suboptimal lipid levels at admission regardless of statin or ezetimibe use. Thus, in a handful of trials that include a large proportion of statin-naïve patients (e.g., EVOPACS), evidence shows that immediate PCSK9 inhibitor initiation is a feasible alternative to the current recommendations. This tactic should have several benefits, not only related to the pleiotropic actions of these agents, as are going to be described below, but also to the increased adherence and decreased dosing frequency observed with these inhibitors. Furthermore, as shown in the majority of trials, the use of PCSK9 inhibitors leads to rapid reductions in LDL-C levels, which are significantly reduced compared to baseline by day 1–3 in each respective study. This is particularly significant not only for normalizing lipid levels and preventing future events but also for plaque stabilization. Despite the positive results from clinical studies, the notion for early PCSK9 inhibitor initiation after ACS is not reflected in the recent 2023 European Society of Cardiology Guidelines on the management of ACS, which state that PCSK9 use after ACS should be suggested during admission only in patients under maximum tolerated high-intensity statin and ezetimibe treatment, with suboptimal LDL-C levels (>55 mg/dL) [63]. On the other hand, PCSK9 inhibition early after the ACS event is mentioned in a 2022 European Clinical Consensus [64], in which it is stated that patients with increased ischemic risk factors (multivessel disease, familial hypercholesterolemia) that are unlikely to achieve LDL-C targets or patients required to achieve LDL-C levels < 40 mg/dL (recurrent ischemic events in less than 2 years) may benefit from the addition of PCSK9 inhibitor early on. Although there is a significant benefit of early PCSK9 inhibitors in ACS, given their pleiotropic actions and the rapid decline in LDL-C levels, there are several limitations in everyday practice that could influence the large-scale use of this strategy. One of them is potentially limited availability due to cost. Analyses show that, even after cost reductions and an increase in use through the years, an important number of eligible patients are not being prescribed this agent due to economic or delivery mode barriers [65,66]. Considering both the benefits and limitations of these agents, further trials, novel protocols, and consensus supporting the early initiation, as well as finding ways to avoid prescription and cost barriers, could validate the role of these agents in ACS in the light of more large-scale evidence.

## 5. PCSK9 Inhibitors in Acute Coronary Syndromes: More than Lipid Lowering

Immediate LDL-C reduction after an acute coronary syndrome (ACS) has been linked, as aforementioned, with significant benefits in mortality and adverse outcomes in both statin and PCSK9 studies in cohorts evaluating immediate initiation of LDL-C lowering treatment. However, as reported earlier in trials using an extensive lipid-lowering strategy with high-intensity statin treatment, the benefits associated with this reduction extend beyond just lipid lowering to plaque modification, endothelial function, and microcirculation. These effects can affect post-ACS coronary circulation, resulting in plaque stability and reduced microcirculatory dysfunction and thus in further enhanced outcomes and potentially improved survival. Several studies have evaluated the effect of PCSK9 inhibitors on plaque characteristics and the endothelium, which are going to be described in the following paragraphs.

### 5.1. PCSK9 and Plaque Modification

Nichols et al. were one of the first groups to evaluate treatment with evolocumab in the GLAGOV study [67]. They included 968 patients observed over a 76-week follow-up. The primary efficacy endpoint was the percentage of atheroma volume, which was calculated using the following equation: PAV = Σ(EEM_area_ − Lumen_area_)/ΣEEM_area_ × 100, where EEM_area_ is the cross-sectional area of the external elastic membrane, and Lumen_area_ is the cross-sectional area of the lumen. A secondary efficacy endpoint, normalized total atheroma volume (TAV), was measured using the following equation: TAV_normalized_ = (EEM_area_ − Lumen_area_)/Number of images in pullback × Median number of images in the cohort. Compared to the placebo, evolocumab resulted in lower LDL-C levels, a significant decrease in PAV, and normalized TAV, with a mean difference of −4.9 mm^3^ (95% CI, −7.3 to −2.5; *p* < 0.001). Similar results in CAD patients have been described by Ota et al. [68], showing significant LDL-C reduction in the PCSK9 group (difference of 59.3 mg/dL, *p* < 0.001), with absolute and normal PAV reduction and LCBI regression being significantly more pronounced in the PCSK9 arm. Notably, the change in LDL-C reduction was significantly associated with plaque regression. Thus, the investigators report that evolocumab resulted in a greater plaque regression compared to placebo, showcasing its effect on plaque modification.

Moreover, the HUGYENS study assessed the effect of evolocumab on plaque characteristics after MI [69]. The study group included patients with NSTEMI, who either received evolocumab or a placebo for 52 weeks on top of statin treatment and evaluated their plaque characteristics with OCT. At follow-up, the evolocumab treatment showed a significant increase in the minimum fibrous cap thickness (+42.7 vs. +21.5 μm; *p* = 0.015), as well as a decrease in maximum lipid arc (−57.5° vs. −31.4°; *p* = 0.04). Moreover, there was a greater regression of PAV with the use of evolocumab (−2.29% ± 0.47% vs. −0.61% ± 0.46%; *p* = 0.009), without, however, any difference in plaque calcium.

The PACMAN-AMI trial [70] investigated the effect of alirocumab on top of statin therapy in atherosclerotic characteristics after MI. A total of 300 patients were enrolled, with 148 receiving alirocumab and 152 receiving a placebo, less than 24 h after PCI. Available imaging modalities included IVUS, OCT, and near-infrared spectroscopy (NIRS) and were used to evaluate plaque features at baseline and at 52 weeks follow-up. In concordance with the aforementioned studies, the administration of alirocumab resulted in a significant mean change in PAV (difference, −1.21% [95% CI, −1.78% to −0.65%], *p* < 0.001), maximum lipid core burden index within 4 mm (LCBI_4 mm_) (difference, −41.24 [95% CI, −70.71 to −11.77]; *p* = 0.006), and minimal fibrous cap thickness (difference, 29.65 μm [95% CI, 11.75–47.55]; *p* = 0.001). No significant difference in adverse events was noted.

Further studies on alirocumab also corroborated the significant regression of plaque vulnerability features with the use of PCSK9 in both stable and acute diseases. In particular, Gao et al. [71] reported a significantly greater reduction in LDL-C levels, associated with a significant increase in fibrous cap thickness and minimum lumen area in patients with intermediate coronary stenoses. Similarly, the ALTAIR study [72], evaluating the initiation of alirocumab post-PCI, showed significantly increased fibrous cap thickness and decreased lipid core and macrophage grade without, however, associated significant changes in the lumen area.

Data on the PCSK9-inhibitor effect on plaque stability are also available in Japanese populations after ACS. The J-IVUS study [73] recruited post-ACS patients with a baseline LDL > 100 mg/dL while on statins or statin-naïve patients with LDL-C targets above normal values, which were randomized to receive either a PCSK9 inhibitor and standard therapy (n = 103) or only standard therapy (n = 103), for a 36-week follow-up period. Normalized TAV and PAV differences at follow-up were numerically higher with PCSK9 inhibitors but not significantly different between the two arms (*p* = 0.23 and 0.79, respectively). Notably, LDL-C reduction was significantly greater with PCSK9 inhibitors. Thus, the non-significant results cannot be explained by insufficient LDL-C reduction. In a similar cohort, however, Yano et al. [74] showed that the prescription of evolocumab in patients with ACS significantly increased OCT-derived fibrous cap thickness and decreased macrophage grade and lipid core in correlation with LDL-C levels’ reduction. Although no differences in PCSK9 inhibitor efficacy have been observed in ethnic groups [49], it is important that future studies should address genomics to understand potential differences in plaque modification post-ACS.

Despite not assessing ACS populations, the ARCHITECT study evaluated PCSK9 inhibitor-associated plaque modification on CCTA features of patients with familial hypercholesterolemia. Perez de Isla et al. [75] assessed 104 patients treated with alirocumab for 78 weeks and compared baseline and follow-up CCTA high-risk plaque characteristics. At follow-up, the coronary plaque burden significantly decreased from 34.6% at baseline to 30.4% (*p* < 0.001), while an increase in the proportion of calcified (+0.3%; *p* < 0.001) and mainly fibrous (+6.2%; *p* < 0.001) plaque and a decrease in the percentage of fibro-fatty (−3.9%; *p* < 0.001) and necrotic plaque (−0.6%; *p* < 0.001) was also noted. Therefore, along with intracoronary imaging, non-invasive evaluation of plaque composition with CCTA could also assist in the evaluation of the presence and extent of plaque regression, being a valuable tool not only in trials but also in clinical practice and patient follow-up.

Aiming to combine the results of these studies and provide a more in-depth understanding of the effect of PCSK9 inhibitors in coronary plaques, Liu et al. [76] performed a meta-analysis, including seven RCTs and two observational trials, with a total of 2290 patients. The addition of PCSK9 inhibition resulted in significantly decreased TAV and PAV, mostly in Caucasians, with the study noting that the effect was unclear in Asians. Thus, despite showcasing the efficacy of PCSK9 inhibition in plaque regression, the authors highlight the need for more studies in general, as well as more investigations focusing on specific populations.

Finally, although the evidence so far shows a benefit of PCSK9 addition in the management plan regarding plaque stabilization parameters, less is known about its effect on survival and outcomes. In this context, in a PACMAN-AMI sub-study, it was reported that a “triple-regression” phenotype, i.e., the combined presence of PAV reduction, LCBI_4 mm_ reduction, and minimal fibrous cap thickness increase, which was independently predicted by treatment with a PCSK9 inhibitor, resulted in a significant reduction in the composite of mortality, MI, and ischemia-driven revascularization [77]. Given the positive effects of PCSK9 inhibitors on plaque characteristics, LDL-C levels, and MACEs, it is largely supported that they should be started early, especially after ACS, in order to maximize benefit and stabilize any prone-to-rupture lesions [78]. A similar indication could be patients with significant high-risk plaque presence, who, as they are at an increased rate of subsequent events, could potentially benefit from plaque regression and, ultimately, prevent ischemia. This suggestion should be properly investigated in well-powered trials in order to provide evidence regarding prophylactic extensive lipid lowering based on imaging criteria.

### 5.2. PCSK9 Inhibition and Microcirculation

Microcirculation and its dysfunction are factors that largely affect cardiovascular disease. It is well known that endothelial dysfunction and inflammation promote dysregulation of vasorelaxation, thus resulting in microvascular disease. Several key molecular pathways are responsible for this pathogenetic process. While the results of PSCK9 inhibitor trials and their effect on prognosis and LDL-C lowering became available, several researchers assessed if inhibition of this enzyme could result in changes in microcirculatory regulation.

Inflammation is a major driver of atherosclerotic disease, even in the early stages of the pathology. Endothelial injury, different molecules (LDL-C and oxidized LDL-C), and endothelial shear stress, either directly or indirectly by scavenger receptors (LOX-1), promote the transmigration of inflammatory cells into the arterial intima and the production of cytokines, thus enhancing systemic and local inflammation [79,80]. PCSK9 is present in atherosclerotic lesions and has been shown to have LDL-receptor-related effects [81,82]. Studies evaluating preclinical models show that PCSK9-inhibitor treatment leads to the regression of inflammation and early atherosclerosis biomarkers, mostly via interfering with the nf-κB factor and eNOS pathways, as well as monocyte adhesion to endothelial cells [83]. Other studies explain these results in relation to LDL-C reduction and lipopolysaccharide (LPS) intracellular concentration, which is increased after PCSK9 treatment and decreased after inhibition, along with a reduction in other inflammatory cytokines (IL-6 and 8 and VCAM-1) [84]. Despite bench studies showing and validating the effect of PCSK9 inhibition in coronary inflammation, this is not depicted in clinical studies. Specifically, an analysis of both stable CAD [85] and ACS [51] patients showed no effect of PCSK9 inhibition in highly sensitive C-reactive protein (hs-CRP), with a meta-analysis also confirming these findings [86]. However, more recent analyses have shown that despite the systemic surrogate of inflammation, hs-CRP does not necessarily decrease, and there is an attenuation of arterial wall inflammation observed with PCSK9 inhibitor treatment [87]. Similar results have been shown by Marfella et al. [88], reporting that treatment with PCSK9 inhibitors reduced the expression of pro-inflammatory proteins and increased the abundance of SIRT3 and collagen in atherosclerotic carotid plaques, compared to patients receiving other lipid-lowering drugs and despite similar hs-CRP levels between the groups. Thus, association with PCSK9 inhibition inflammation reduction may be localized and not expressed in a systemic manner. Further studies evaluating the anti-inflammatory effects of these agents at the coronary artery level are highly necessary in order to clarify this complex relation.

Endothelial dysfunction is a well-recognized culprit of coronary microvascular disease (CMD), as well as atherosclerosis. Given the effects of PCSK9 inhibition in platelet and thrombus formation, as will be described below, and in atherosclerosis pathogenesis, it is possible that these agents also influence endothelial and promote its regression. Preclinical models have shown the ability of PCSK9 inhibitors to reduce oxidative stress, autophagy, and overall dysfunction via several molecular pathways, including NAD-dependent deacetylase sirtuin-3 and 4 (SIRT3 and SIRT4) [89,90,91]. However, the clinical results are conflicting. Schremmer et al. [92] showed that PCSK9 inhibitors at 6 months follow-up significantly increased flow-mediated dilation (FMD), decreased aortic augmentation index and increased peripheral tissue oxygenation in the overall cohort, highlighting the positive effects of PCSK9 in endothelial dysfunction and microcirculation. Similar results in microcirculation were noted by Ji et al. in NSTEMI patients, with a significant decrease in the index of microvascular resistance (IMR) [93]. However, when compared to statin treatment, a prespecified secondary analysis of the PACMAN-AMI study found no difference in FMD at 52 weeks follow-up, which was similarly increased in both cohorts [94], while regarding CMD, the EVOCATION trial [95], despite also noting significant LDL-C reductions, did not result in significant changes in IMR or major periprocedural MI events.

### 5.3. PCSK9 Inhibition and Platelet Function

Thrombosis is an important consideration for patients after an ACS, with the addition of antiplatelet agents playing a pivotal role in the post-interventional management of MI in order to reduce both short- and long-term ischemic events [96]. An important aspect of thrombotic phenomena in both ACS and stable disease is platelet reactivity, which is associated with mortality, MI, and stent thrombosis after PCI [97]. Several parameters have been shown to affect platelet reactivity, including social factors (smoking), concomitant pathologies (diabetes), drug–drug interactions, and genetics [98,99]. This led researchers to further investigate factors influencing platelet reactivity, including studies evaluating the role of PCSK9. The association of PCSK9 levels and platelet reactivity has been shown in both patients with stable [100] and acute [101] disease, as well as in vitro studies without the influence of antiplatelet or statin treatment [102] and in PCSK9-knockout mice [103]. Interestingly, PCSK9 in vivo has been shown not only to increase platelet activation but also to expand the ischemic lesion post-MI in animal models [104].

Only limited data are available to date evaluating PCSK9 inhibitor use in platelet reactivity. Franchi et al. [105], in their randomized study, showed that in patients with atherosclerotic cardiovascular disease, evolocumab, in addition to statin therapy, did not significantly reduce platelet reactivity units (PRU) at 30 days in the total cohort (mean difference 22 PRUs in the high platelet reactivity arm). However, they were significantly reduced in the high platelet reactivity (HPR) sub-analysis at 14 days (218.2 ± 29.7 vs. 246.6 ± 35.2; *p* = 0.017, mean difference 28 PRUs). Ziogos et al. [106], however, showed that evolocumab treatment significantly reduced platelet factor 4 and von Willebrand factor serum levels at 30 days, compared to the placebo, in ACS patients. Providing more data on PRUs, a sub-study of the PACMAN-AMI trial [107] indicated that alirocumab treatment, in ACS patients receiving dual antiplatelet therapy, resulted in no significant difference in PRU at 4 weeks (12.5 [IQR: 27.0] vs. 19.0 [IQR: 30.0], *p* = 0.26), regardless of high-potency antiplatelet treatment. These results are of interest, as despite in vivo trials documenting the association of PSCK9 with platelet reactivity, clinical trials report only a modest effect of treatment in platelet reactivity, which is of unknown clinical significance. As modulating platelet reactivity with PCSK9 inhibitors could have a substantial effect, especially in HPR and high-bleeding risk patients, investigators should further examine these pathophysiological relationships and provide more answers regarding any platelet-inhibitory effect of these agents.

## 6. Future Directions

**Figure 1:**
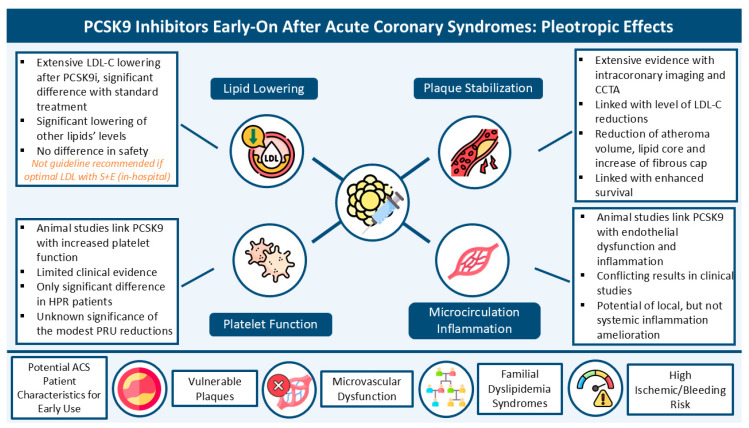
PCSK9 inhibitors early on after acute coronary syndromes: pleiotropic effects. Abbreviations: S: statin; E: ezetimibe; PCSK9: Proprotein Convertase Subtilisin–Kexin Type 9; PCSK9i: Proprotein Convertase Subtilisin–Kexin Type 9 Inhibitor; LDL-C: low-density lipoprotein—cholesterol; HPR: high platelet reactivity; PRU: platelet reactivity unit; CCTA: Coronary Computed Tomography—Angiography.

Early initiation of PCSK9 inhibitors after ACS is an appealing method for immediate, intensive lipid lowering, which, as shown by clinical trials, is safe and efficient in achieving post-MI lipid targets. More important, however, are the potential pleiotropic effects of PCSK9 inhibitors (Figure 1). Along with the well-documented effect on lipid levels, both in LDL-C as well as other pro-atherogenic lipoproteins, these agents have been related to improved atherosclerotic plaque features, resulting in plaque stabilization or regression and survival benefit, as well as inflammation and platelet reactivity reduction. Regarding the latter two, evidence is not as concrete as in other PCSK9 effects, given the small amount of evidence and some neutral results. Thus, further research is needed with respect to the pleiotropic effects of these agents, as well as more extensive investigation and long-term follow-up regarding survival and cardiovascular adverse events. This research frontier could alter the current state of lipid-lowering prescription after MI and change the current clinical practice, which suggests that the prescription of PCSK9 inhibitors during admission only in patients already under a high-intensity statin and ezetimibe treatment and not on LDL-C targets (<55 mg/dL) [63] toward an early PCKS9 inhibitor use, especially in phenotypes, which would ultimately benefit the most, potentially after addressing the aforementioned barriers of large-scale PCSK9 inhibitor use. Such potential phenotypes, considering the aforementioned pleiotropic effects, could be patients with identified high-risk (vulnerable) atherosclerotic coronary plaques, microvascular dysfunction, high ischemic risk, and familial dyslipidemia disorders, among others yet to be identified and investigated.

As aforementioned, PCSK9 inhibitors could have an anti-inflammatory effect. Anti-inflammatory therapy in patients with MI is a relatively novel hypothesis, which aims to target the residual inflammatory burden of ACS patients already on optimal management [108]. Except for PCSK9 inhibitors, other agents, such as colchicine, are tested for secondary prevention. The results from the COLCOT trial showed that treatment with 0.5 mg of colchicine reduced ischemic cardiovascular events at a mean of 22 months follow-up after ACS, mostly related to stroke and angina requiring revascularization rate reduction [109]. However, similar randomized studies (COPS and COLCHICINE-PCI trials) did not show any benefit of colchicine in cardiovascular outcomes, with a notably higher mortality rate in the colchicine arm, mostly due to non-cardiovascular death [110] or PCI-related myocardial injury when given pre-procedurally [111]. However, maybe a more extended follow-up is needed, as the two-year follow-up of the COPS study [112] showed a significant reduction in the composite of mortality and ischemic events, with the signal of increased non-cardiovascular mortality still remaining. More studies on anti-inflammatory agents [113], as well as PCSK9 inhibitors, would provide important insight into the modulation of inflammation and cardiovascular outcomes after ACS.

Finally, novel PCSK9 modulatory agents may also have a role in the management of the ACS patient. Inclisiran is a novel, subcutaneously administered, first-in-class drug that acts as a small interfering RNA (siRNA). Its main mechanism of action is the prevention of the synthesis of PCSK9 at the hepatic level [114]. Inclisiran requires less frequency than monoclonal antibody regimen administration and has similar safety and efficiency in reducing LDL-C and PCSK9 levels, as shown in studies for both primary and secondary prevention and familial hypercholesterolemia [115,116]. Recently, a post hoc pooled analysis of the ORION-10 and ORION-11 randomized trials regarding patients with previous MI [117] showed that inclisiran was safe and effective regarding LDL-C lowering (reduction from baseline: 52.6% in those with MI < 1 year and 50.4% in those with MI > 1 year), independently of the timeframe of MI history. To date, no trials have evaluated the effect of inclisiran solely in ACS patients, as well as post-ACS initiation of the agent. Further research on this topic would provide much-needed evidence and an alternative to the currently available PCSK9 inhibitors, enhancing the options for optimally managing post-MI patients.

## 7. Conclusions

Early PCSK9 inhibition in patients with ACS is a safe and feasible strategy, resulting in pronounced and rapid LDL-C and other lipid level reduction. Along with the associated benefit of intensive lipid lowering, the pleiotropic effects of these agents in atherosclerotic plaque stability, endothelial function, microcirculation, inflammation, and platelet reactivity make these inhibitors of particular interest in the post-ACS timeframe, potentially extending the benefit of lipid lowering in a multifactorial effect. Future large-scale studies are still necessary in order to provide more evidence, both in survival, adverse outcomes, and pleiotropic effects of PCSK9 inhibitors, so as to inform clinical practice guidelines in the near future.
